# The Role of IL-33 in Host Response to *Candida albicans*

**DOI:** 10.1155/2014/340690

**Published:** 2014-07-21

**Authors:** C. Rodríguez-Cerdeira, A. Lopez-Bárcenas, B. Sánchez-Blanco, R. Arenas

**Affiliations:** ^1^Department of Dermatology, Hospital do Meixoeiro (CHUVI) and University of Vigo, C/Meixoeiro S/N, Vigo, 36200 Galicia, Spain; ^2^Department of Dermatology, Hospital General Dr. Manuel Gea González, Calzada de Tlalpan 4800, Tlalpan, 14000 México City, DF, Mexico; ^3^Department of Emergency, CHUVI, Hospital do Meixoeiro (CHUVI), C/Meixoeiro S/N, Vigo, 36200 Galicia, Spain; ^4^Department of Dermatology (Section of Mycology), Hospital General Dr. Manuel Gea González, Calzada de Tlalpan 4800, Tlalpan, 14000 México City, DF, Mexico

## Abstract

*Background*. Interleukin (IL) 33 is a recently identified pleiotropic cytokine that influences the activity of multiple cell types and orchestrates complex innate and adaptive immune responses. *Methods*. We performed an extensive review of the literature published between 2005 and 2013 on IL-33 and related cytokines, their functions, and their regulation of the immune system following *Candida albicans* colonization. Our literature review included cross-references from retrieved articles and specific data from our own studies. *Results*. IL-33 (IL-1F11) is a recently identified member of the IL-1 family of cytokines. Accumulating evidence suggests a pivotal role of the IL-33/ST2 axis in host immune defense against fungal pathogens, including *C. albicans*. IL-33 induces a Th2-type inflammatory response and activates both innate and adaptive immunity. Studies in animal models have shown that Th2 inflammatory responses have a beneficial role in immunity against gastrointestinal and systemic infections by *Candida* spp. *Conclusions*. This review summarizes the most important clinical studies and case reports describing the beneficial role of IL-33 in immunity and host defense mechanisms against pathogenic fungi. The finding that the IL-33/ST2 axis is involved in therapeutic target has implications for the prevention and treatment of inflammatory diseases, including acute or chronic candidiasis.

## 1. Introduction

*Candida* spp. are dimorphic fungi that commonly colonize multiple tissues and organs, including the kidney, brain, oral and vaginal mucosa, skin, and gastrointestinal (GI) tract of adult humans. Their overgrowth is prevented by competing commensal bacteria, as well as local host immune responses. However, perturbations of the normal flora by antibiotic treatment or immunocompromised states may lead to the overgrowth of* Candida* spp. on mucosal surfaces, resulting in the progression of oropharyngeal candidiasis (also known as thrush).* Candida albicans* has been identified as the leading cause of fatal fungal infections, with mortality rates as high as 50%, and is the fourth most common cause of bloodstream and nosocomial infections [1–4].

Host recognition of* Candida* spp. requires the engagement of surface receptors on innate immune cells, including toll-like receptor (TLR) 2 and Dectin-1 [5, 6]. A major consequence of receptor activation is the induction of proinflammatory gene expression. This leads to production of the zymogen interleukin 1 beta (IL-1*β*), which is proteolytically processed by caspase-1, rendering it biologically active. Activation of caspase-1 requires signaling through inflammasomes, which are protein complexes consisting of either nucleotide-binding oligomerization domain-like receptors (NLRs) or pyrin and HIN domain-containing protein, which is absent in melanoma-2 [7–11]. NLRs are characterized by the presence of a leucine-rich repeat domain, a central NACHT domain involved in oligomerization and protein-protein interactions, and a caspase activation and recruitment domain (CARD) or pyrin domain. Upon exposure to activating stimuli, NLRs undergo conformational changes, facilitating their oligomerization with apoptosis-associated speck-like protein containing a CARD. This in turn leads to autocatalytic cleavage of procaspase-1 and its activation, resulting in the subsequent cleavage of pro-IL-1*β*. Several intracellular danger signals and crystalline compounds such as uric acid crystals [12], cholesterol crystals, amyloid, and asbestos have been shown to activate the NACHT, LRR, and PYD domain-containing protein (NLRP3) inflammasome [13–15], but the precise mechanism underlying inflammasome activation has yet to be elucidated. Several possible mechanisms have been proposed, including mitochondrial reactive oxygen species production, phagosomal or endosomal rupture, and cell membrane disturbances. The NLRP3 [16] inflammasome has been shown to be associated with the IL-1*β* response to pathogen-derived molecules such as bacterial muramyl dipeptide and toxins with responses to a range of bacterial, viral, and fungal pathogens, including* C. albicans* [17, 18]. Similarly, another NLR, NLRC4, forms an inflammasome capable of activating caspase-1, resulting in IL-1*β* cleavage [19].

**Figure 1:**
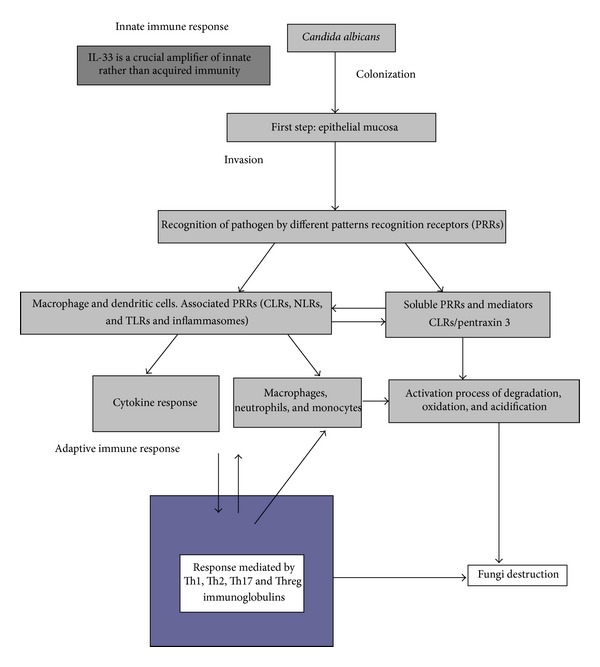
Pathways of host immune response to Candida colonization. CLRs, NLRs, and TLRs. CLRs (C-type lectin receptors); NLRs (NODE-like receptors); and TLRs (toll-like receptors) (modified from [21]).

The adaptive immune response of the host must also be considered. Dendritic cells are an important link between innate and adaptive immunity. The type of immune response in these cells largely depends on the morphological characteristics of* C. albicans* [20]. Yeast cells and pseudohyphal cells interact with dendritic cells via different receptors and thus elicit different responses. Dendritic cells that phagocytize the yeast form of* C. albicans* induce differentiation of CD4^+^ cells into Th1 cells, while dendritic cells stimulated by the pseudohyphal form induce a Th2 response. The response mediated by Th1 cells is associated with protection of the host against fungal infection. The Th2 response is related to the ability of microorganisms to avoid or suppress the host's immune response. Regardless, the result of either Th response is the activation of B cells and the maturation of other phagocytic cells (Figure 1) [21, 22].

Candidiasis manifests as a number of clinical conditions that have been extensively discussed elsewhere. Our understanding of host responses to this pathogen is incomplete; however, as depicted in Figure 1, emerging data indicate that the production of key cytokines such as IL-12 and IL-23 by the innate immune system is essential for the initiation of appropriate adaptive responses in mucocutaneous disease [21]. Recently, IL-33 (also known as IL-1F11), which is homologous to IL-18, was identified as a new member of the IL-1 family, which comprises IL-1*α*, IL-1*β*, IL-18, and IL-1Ra. It has been reported that IL-33 plays an important role in innate and adaptive immunity [23].

This paper presents an overview of the role of IL-33 in systemic and mucosal infections and its relationship with other inflammatory factors and interleukins during an immune response.

## 2. Methodology

We performed an extensive search of the Cochrane Central Register of Controlled Trials, MEDLINE (PubMed), and Embase for articles published from 2005 to January 2012. We also examined references from selected articles. We included case series with five or more patients, cohort studies, and randomized controlled trials. The search terms included* C. albicans*, candidiasis, IL-1*α*, IL-1*β*, IL-18, IL-1Ra, IL-33, TLR, and IL-33/ST2 axis. We also included some data from our own studies.

## 3. Results and Discussion

### 3.1. Biology

IL-33 is an important member of the IL-1 family, which has multiple activities in innate and adaptive immune responses. IL-33 signaling is mediated through the receptors ST2 and IL-1 receptor accessory protein (IL-1RAcP), both of which are members of the IL-1 receptor (IL-1R) family. Combined crystallography and small-angle X-ray scattering studies revealed that ST2 possesses flexibility in the hinge region between the D3 domain and the D1D2 module, whereas IL-1RAcP exhibits a rigid conformation in the unbound state in solution. The molecular flexibility of ST2 provides structural insights into domain-level conformational changes of IL-1 primary receptors upon ligand binding, and the rigidity of IL-1RAcP explains its inability to bind ligands directly. The architecture of the IL-33/ST2/IL-1RAcP complex in solution, obtained by small-angle X-ray scattering analysis, resembles that of IL-1*β*/IL-1RI/IL-1RAcP and IL-1*β*/IL-1RII/IL-1RAcP crystal structures. Collectively, these results reveal IL-33 structure-function relationships, supporting and extending a general model for ligand-receptor assembly and receptor activation in the IL-1 family [24].

**Figure 2:**
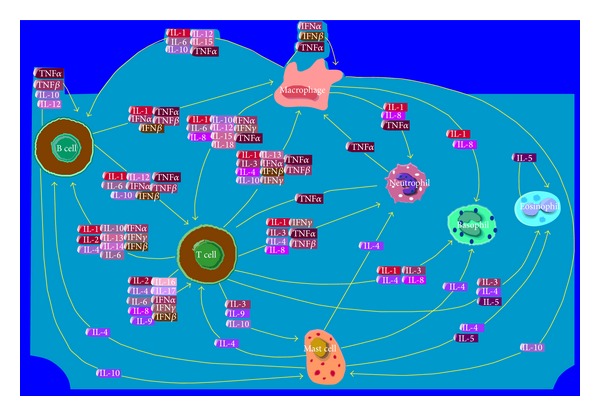
IL-1F11 is a recently identified member of the IL-1 family cytokines which stimulate a broad inflammatory response and promotes the recruitment of neutrophils to the site of infection. IL-1F11 = IL33.

IL-33 has been detected in epithelial cells (source of IL-1 in normal and diseased epithelium) (Figure 2) [25], fibroblasts, and endothelial cells in inflammatory tissue of patients with rheumatoid arthritis and Crohn's disease. IL-1*β* is produced by epithelial cells in large quantities and is localized in the superficial layers of the epithelium as an inactive precursor of 31–33 kDa. The precursor, pro-IL-1*β*, is located in cell nuclei and is released by necrotic cells, triggering an immune response. Western blotting analysis of IL-1*β* in epithelial cells showed a significant increase in the amount of this precursor during the initial stages of an infection by* Candida* spp. and a decrease through subsequent stages.

#### 3.1.1. Immunobiology

IL-33 binds to ST2L (the membrane-bound form of ST2 and a member of the TLR/IL-1R superfamily), forming a functional complex (IL-33/ST2L) that then binds to IL-1RAcP [26–28]. Signaling induced through the cytoplasmic toll-interleukin 1 receptor domain of IL-1RAcP leads to recruitment of an adaptor protein, myeloid differentiation marker 88, and activation of the transcription factor NF-*κ*B via TNF-*α* receptor associated factor 6, IL-1 receptor associated kinase 1/4, and mitogen-activated protein kinase, which in turn leads to the production of inflammatory mediators. This ST2L-mediated signaling is negatively regulated by SIGIRR (single immunoglobulin IL-1R-related molecule), resulting in suppression of the immune response [29].

### 3.2. Role in Pathogenesis

IL-33 has a dual role, acting as a traditional cytokine through receptor activation and as an intracellular nuclear transcriptional regulator through formation of a complex with ST2L. Recent studies suggest that proteolytic processing may not be a prerequisite for the functioning and biological activation of the IL-33/ST2L signaling pathway, as IL-33 lacking a caspase-1 splice site is biologically active. This is further evident from the fact that caspases targeting IL-33 lead to the inactivation of IL-33 and its proinflammatory properties [30–32]. It is presumed that the biologically active full-length IL-33 is released during necrotic damage or apoptosis as a cellular signal [33].

During host defense against pathogens, innate immune cells recognize “pathogen-associated molecular patterns” directly via TLRs, resulting in the induction of local or systemic inflammation. In addition, during trauma or infection, necrotic cells release endogenous proinflammatory factors referred to as “damage-associated molecular patterns” (DAMPs; also called “alarmins”), which provoke and promote a local and/or systemic inflammatory response. For example, high-mobility group box 1 (HMGB1), which was initially identified as a nuclear transcriptional regulatory factor, is released by macrophages in response to lipopolysaccharide (LPS) and induces inflammation [33]. Several recent studies have demonstrated that IL-33, like HMGB1, localizes in the nucleus and may also act as a DAMP or alarmin. Schmitz et al. [34–36] demonstrated that among the members of the IL-1 family of cytokines, IL-33 shares the highest amino acid sequence homology with IL-18. Like IL-1*β* and IL-18, IL-33 lacks a signal sequence at the N-terminus and is therefore not secreted via the conventional vesicle transport pathway. Additionally, and again like IL-1*β* and IL-18, IL-33 is reported to be cleaved from pro-IL-33 by caspase-1* in vitro*, suggesting that IL-33 may be secreted following activation of NLRP3 inflammasomes. However, pro-IL-33 lacks the typical cleavage site that exists in pro-IL-1*β* and pro-IL-18. The proteolytic cleavage of pro-IL-33 by caspase-1 occurs at the cytokine motif and not in the intermediate region between the helix-turn-helix and cytokine motifs, which leads to the inactivation of IL-33. Notably, caspase-3 and caspase-7 are similar in function to caspase-1 and cleave pro-IL-33 during apoptosis, and IL-33 processed by these caspases does not express biological activity via ST2/IL-1RAcP; moreover, the apoptotic cells involved do not cause inflammation. On the other hand, it is known that pro-IL-33 is released by necrotic cells without any proteolytic processing by caspases (1, 3, 7, and 8) or calpain 4. In addition, unlike pro-IL-1*β*, pro-IL-33 has biological activity and can induce mouse mast cell activation, which further leads to the production of cytokines via ST2/IL-1RacP-mediated signaling. These observations suggest that pro-IL-33 released by necrotic cells during tissue injury may play a DAMP/alarmin-like role in the induction of inflammation. However, it remains unclear whether IL-33 can act as a potent adjuvant and effectively promote acquired immune responses compared with other alarmin adjuvants such as ATP, which is also released during necrosis [37].

Unlike other members of the IL-1 family, IL-33 primarily induces a Th2 immune response in many immune cells [38], although there is increasing evidence that it may play a role in acute and chronic inflammation. Initially, it was reported that ST2L was selectively expressed by Th2 but not Th1 or regulatory T cells [39]. However, subsequent studies have shown that IL-33 can activate murine dendritic cells directly through the polarization of naive T cells (innocent or Th0) to the Th2 phenotype. IL-33 may act directly on Th2 cells, enabling increased secretion of cytokines, such as IL-5 and IL-13, and may also act as a chemoattractant for Th2 cells [40].

IL-33 is a potent activator of cells of the innate immune system. It activates mast cells and basophils, inducing their degranulation, maturation, and survival and thereby promoting their ability to produce multiple proinflammatory cytokines. IL-33 also has a crucial role in amplifying the inflammatory response via the regulation of CXCR2 and CR3 expression in neutrophils [41, 42]. IL-33 may be a positive or negative regulator of dendritic cells. Cultured bone marrow-derived dendritic cells produce proinflammatory cytokines and upregulate cell surface molecules in response to IL-33. Likewise, IL-33-primed dendritic cells can efficiently induce allergic airway inflammation [43].

Neutrophils represent the largest group of initially recruited phagocytes at the site of* C. albicans* infection. Neutrophils moderately express the receptors TLR2, TLR4, and Dectin-1 and strongly express phagocyte receptors such as complement receptor 3 (CR3, also known as Mac-1, CD11b/CD18, or integrin*α*_M_ *β*_2_) and Fc*γ* receptors (Fc*γ*Rs). Except for Fc*γ*Rs, these receptors can directly recognize the components of the cell wall of* C. albicans* and transduce signals leading to a broad range of biological activities. Dectin-1 signaling plays a critical role in controlling* C. albicans* infection by promoting phagocytosis and the fungicidal action of macrophages and inducing the production of cytokines by macrophages and dendritic cells. In neutrophils, IL-33 blocks the downregulation of CXCR2 and inhibits the activation-induced chemotaxis of TLR4 [44]. In macrophages constitutively expressing ST2L, IL-33 may mediate guided polarization to an alternative active form or M2 phenotype, which enhances Th2 immune responses, thereby improving the LPS-induced production of TNF-*α* in these cells [36, 40, 45].

CR3-mediated signals are involved in the activation of neutrophils and their functions, including firm adhesion, migration, phagocytosis, and killing. CR3 expression is rapidly regulated by a number of stimulators such as granulocyte-macrophage colony-stimulating factor (GM-CSF), formyl-methionyl-leucyl-phenylalanine, TNF-*α*, and IL-10, and upregulation of CR3 expression is important in the migration of neutrophils through venules during inflammation [40]. The CR3 of neutrophils recognizes opsonized and nonopsonized* C. albicans*, and the CR3 signaling pathway mediates the cellular events causing the death of this pathogen [40].

The locations of a lesion, the interleukins at the site, and the functional relationships among interleukins are important determinants of immune responses. For example, in the oral cavity, IL-12 is thought to be at the center of immune responses, and its role has been reevaluated in the light of the functions of IL-23. However, the relationship between these interleukins has been somewhat overshadowed by the pivotal role of Th17 cells and their cytokines in oral candidiasis.

The human GI tract, with its epithelial barrier consisting of a total surface area of approximately 200 m^2^, is the organ most exposed to the external environment. The intestinal barrier is a functional unit responsible for two main tasks crucial for survival of the individual: nutrient absorption and defense of the body against penetration of unwanted, often dangerous, macromolecules. The gut mucosa is a multilayered system consisting of an external “anatomical” barrier and an inner “functional” (immunological) barrier. Commensal gut microbiota, a mucous layer, and the intestinal epithelial monolayer constitute the anatomical barrier. The deeper inner layer consists of a complex network of immune cells organized in a specialized and compartmentalized system known as “gut-associated lymphoid tissue” (GALT). GALT contains both isolated and aggregated lymphoid follicles and is one of the largest lymphoid organs, containing up to 70% of the body's total number of immunocytes. It is involved in immunity to pathogenic microorganisms and tolerance to commensal bacteria. The ability of GALT to interact with luminal antigens rests on specific mucosal immune cells (i.e., dendritic cells and M cells), primarily localized to Peyer's patches within the ileum, which are intimately positioned at the mucosal-environmental interface and internalize microorganisms and macromolecules. These specialized immune cells have the ability to present antigens to naïve T lymphocytes, which subsequently produce cytokines and activate mucosal immune responses when needed. The interaction of these components maintains the delicate equilibrium of intestinal homeostasis. Many factors can alter this balance, including alterations in the gut microflora, modifications of the mucus layer, and epithelial damage, leading to increased intestinal permeability and translocation of luminal contents to the underlying mucosa. The integrity of these structures is necessary for the maintenance of normal intestinal barrier function, and dysregulation of the aforementioned components has been implicated in the pathogenesis of not only inflammatory bowel disease but also many other disorders of the GI tract, including infectious enterocolitis, irritable bowel syndrome, small intestinal bowel overgrowth, and allergic food intolerance [46–49].

Kinetic analyses of vaginal infection with* C. albicans* in C57BL/6 mice demonstrated that fungal burdens remained constant throughout the observation period, while levels of polymorphonuclear leukocytes (PMNs), the protein S100A8, and IL-1*β* in vaginal lavage fluid increased from day 3 onward. Lactate dehydrogenase activity was also positively correlated with increased effectors of innate immunity. Additionally, immunodepletion of neutrophils in infected mice confirmed a nonprotective role for PMNs during vaginitis [50].

Emerging evidence indicates a primary role for IL-33 in the maintenance of gut mucosa homeostasis. Like IL-1*α*, IL-33 appears to serve a dual function. Full-length, unprocessed IL-33 contains a nuclear localization sequence and a DNA-binding domain that is constitutively localized to the nucleus of epithelial and endothelial cells. Under normal conditions, IL-33 can act as an intracellular nuclear factor. In response to inflammatory stimuli, IL-33 is released from cells through a currently unknown mechanism, and it behaves as a functional, secreted cytokine. One of the earliest observed biological activities of IL-33 was its ability to promote epithelial proliferation and mucus production, which are obvious functions involved in epithelial restitution and repair, as well as overall mucosal wound healing and protection. Increasing evidence indicates that IL-33, like IL-1*α*, functions as a prototypic alarmin passively released upon cellular damage, stress, or necrosis and is able to serve as a danger signal to alert the immune system of a local threat such as trauma or infection [51, 52]. In this context, IL-33 has the ability to signal local, innate immune responses to mount an effective inflammatory reaction and restore normal gut homeostasis [53].

### 3.3. Implications for Therapy

IL-33 may have merit for systemic antifungal treatment. (1) This cytokine promotes myelopoiesis in the bone marrow through direct action on bone marrow progenitors primed with stem cell factor and FMS-like tyrosine kinase 3 receptor ligand. These actions are antagonistic to those of IL-5 and are mediated in part by GM-CSF [54]. (2) IL-33 rapidly induces migration of neutrophils from the bone marrow to peripheral tissues through increased production of the chemokine CXCL2 by vessel endothelial cells. (3) IL-33 stimulates tissue macrophages to release CXCL1/2 in response to fungal infection. (4) IL-33 acts directly on neutrophils and elevates their migratory, phagocytic, and killing activities. These properties of IL-33 may be advantageous for enhancing innate immunity in immunocompromised patients [39, 42, 55].

In a comprehensive review, Sattler et al. concluded that IL-33 had beneficial effects during host defense against fungal infections. Administration of exogenous IL-33 to mice infected with* C. albicans* induced rapid fungal clearance and reduced mortality. In this case, IL-33 enhanced the phagocytic activity of neutrophils by enhancing their recruitment, reversing TLR-induced CXCR2 downregulation, and activating TLR and Dectin-1 signaling pathways [56].

## 4. Conclusions

IL-33 plays a crucial role in host defense against pathogens. Inflammation is critical in mediating the host response to injury or infection, and IL-33 plays an important role through induction, progression, and maintenance of the inflammatory response. IL-33 is a key cytokine in Th2-mediated host defense, controls the immune response of tissue barriers such as skin and intestine, and activates the innate and adaptive immune systems. Moreover, experimental models show that the Th1 response is associated with resistance to gastrointestinal and systemic infections by* Candida* spp., while the Th2 response is associated with susceptibility to infection. According to the current research findings, manipulation via IL-33/ST2 represents a promising therapeutic strategy for the prevention and treatment of various inflammatory disorders.
